# Exosome-like vesicles released from ob/ob mouse adipose tissue enhance cell survival of cells with radiation-induced genomic instability

**DOI:** 10.1093/jrr/rrac102

**Published:** 2023-01-20

**Authors:** Kentaro Ariyoshi, Yohei Fujishima, Valerie Swee Ting Goh, Akifumi Nakata, Kosuke Kasai, Mitsuaki A Yoshida, Tomisato Miura

**Affiliations:** Integrated Center for Science and Humanities, Fukushima Medical University, 1 Hikariga-oka, Fukushima, Fukushima, 960-1295, Japan; Department of Risk Analysis and Biodosimetry, Institute of Radiation Emergency Medicine, Hirosaki University, 66-1 Hon-cho, Hirosaki City, Aomori 036-8564, Japan; Department of Radiobiology, Singapore Nuclear Research and Safety Initiative, National University of Singapore, Singapore 138602, Singapore; Faculty of Pharmaceutical Sciences, Hokkaido University of Science, Sapporo City, Hokkaido 006-8585, Japan; Department of Bioscience and Laboratory Medicine, Hirosaki University Graduate School of Health Sciences, Hirosaki City, Aomori 036-8564, Japan; Institute of Chromosome Life Science (ICLS),, Fujimino City, Saitama 356-0031 Japan; Department of Risk Analysis and Biodosimetry, Institute of Radiation Emergency Medicine, Hirosaki University, 66-1 Hon-cho, Hirosaki City, Aomori 036-8564, Japan

**Keywords:** radiation-induced genomic instability, extracellular vesicles, adipose tissue, animal model

## Abstract

Multiple epidemiological studies have shown that obesity is a serious risk factor for cancer development. While the underlying mechanisms between obesity and cancer are still unknown, obesity disrupts the role of adipocytes in energy homeostasis, and the alteration of adipokine, insulin and sex steroid signaling. Recently, it has been identified that adipose tissue-derived exosome-like vesicles (ELVs) regulate metabolic homeostasis. In this study, we collected ELVs from adipose tissue of an obese mouse (ob/ob) strain and control mouse (C57BL/6) strain, and checked whether adipose ELVs influence radiation-induced cell death on mouse fibroblast cells (m5S). Furthermore, we analyzed the micronucleus (MN) frequency in survived cells after radiation exposure to investigate the effect of ELVs on radiation-induced genomic instability. We first observed that ELVs from control and obese mice showed enhanced colony forming ability in un-irradiated m5S cells. However, enhanced survival was observed only in 3 Gy-irradiated m5S cells with obese ELV treatment. Despite no ELV effect on colony size, interestingly, the frequency of MN in survived m5S cells after 3 Gy irradiation was elevated when treated obese ELVs compared to control ELVs. These results suggested that obese mouse adipose ELVs could enhance the survival of irradiated cells harboring increased radiation-induced genomic instability.

## INTRODUCTION

Obesity is one of the risk factors of cancer in various organs [1, 2]. The possible mechanism link between obesity and cancer has been implicated by chronic inflammatory state metabolic dysfunction in cancer promotion, epithelial-to-mesenchymal transition and genomic instability [3, 4]. Interestingly, in a recent study, Zhu *et al.* reported that exosome-like vesicles (ELVs) secreted by adipose tissue stem cells reduced the apoptosis of osteocytes after low-level laser irradiation [5]. Moreover, Jeurissen *et al.* reported that ELVs released from cancer-associated adipose tissue promotes stimulation of CREB transcription factor phosphorylation and sphere formation in breast cancer cells [6]. Cell–cell communication between cancer cells and adipose tissue via ELVs have also been discussed [7, 8]. However, little is known about the role of ELVs in carcinogenesis. In this study, we examined ELVs’ effect on radiation-induced genomic instability in a mouse fibroblast cell line m5S with stable karyotype [9]. We demonstrated that the colony forming ability of irradiated m5S was higher after treatment of ELVs from ob/ob mouse adipose tissue. Furthermore, micronucleus (MN) frequency, a DNA damage biomarker [10], in survived m5S cells was elevated. These results suggest that ELVs from adipose tissue in obese mice could serve as radio-protective factors for cells with genomic instability after radiation.

## MATERIALS AND METHODS

### Animals

Eight-week-old wild-type C57BL/6 male and leptin-deficient ob/ob male mouse were purchased from Charles River Japan Inc. The mice were housed in three to an autoclaved cage. Mice were fed with a standard diet (MB-1) and given water ad libitum.

### ELV collection from mouse adipose tissue and labeling

Wild-type C57BL/6 (*n* = 4) and ob/ob (*n* = 3) mouse visceral adipose tissue were collected and cultured separately for 6 hours in DMEM medium at 37°C, 5% CO_2_. ELVs collected from culture medium were isolated using the exoEasy Maxi Kit (Qiagen) according to the manufacturer’s instructions, and the total protein content of ELV was determined using a Qubit 3.0 Fluorometer (Invitrogen) according to the manufacturer’s protocol. The detection of ELV protein by Western blot was described previously [11]. ELVs were labeled with PKH67 labeling kit (Sigma-Aldrich) to check for the ELV cell uptake. Labeled ELVs were added to the cell medium of m5S cells (final concentration: 2 or 4 μg/mL). After 6 hours incubation, cells were fixed with 4% formaldehyde for 15 min, then mounted with Vectashield with DAPI (Vector Laboratories). Images were captured using a fluorescent microscope and CCD camera (Olympus) with the total magnification ×400.

### Cell culture and irradiation

m5S cells were cultured in 5 mL of DMEM supplemented with 10% exosome-depleted fetal bovine serum (SBI), 100 U/mL penicillin, and 100 μg/mL streptomycin (Thermo Fisher Scientific), in a T25 flask at 37°C, 5% CO_2_. Cells were irradiated with an X-ray generator (MBR-1520R-3; Hitachi Medical) at 0 or 3 Gy (1 Gy/min).

### Colony formation assay and MN analysis

**Fig. 1:**
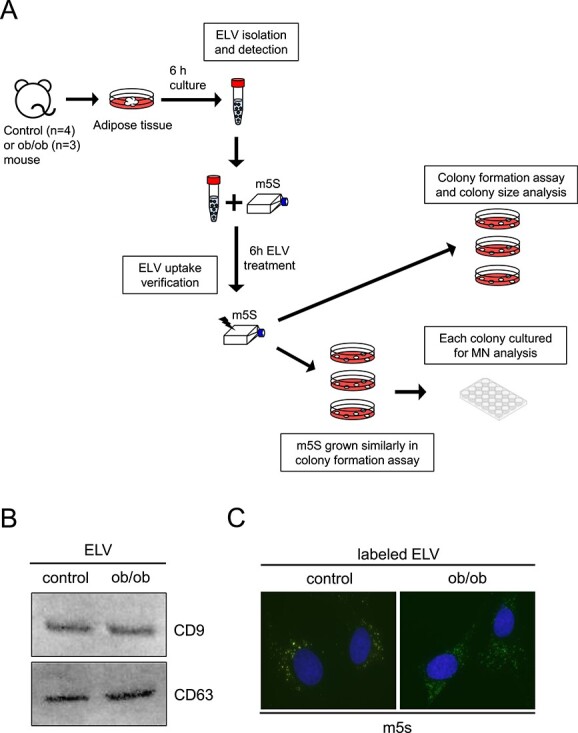
Treatment of ELVs released from mouse adipose on mouse fibroblast cell line, m5S. (A) A schematic view of the experimental protocol. (B) Detection of CD9 and CD63 proteins in ELVs from control and ob/ob mouse adipose tissues by Western blot. (C) Representative images of PKH67-labeled ELV uptake in m5S cells. Nuclei were counterstained with DAPI. Scale bar: 10 μm.

A schematic of the experimental procedure for the colony formation assay is shown in Fig. 1A. Isolated ELVs from adipose tissue of each animal were added to m5S cell medium (final concentration: 2 or 4 μg/mL; m5S grown in 100 mm dishes x 3). Six hours after ELV treatment, m5S cells were irradiated with 0 or 3 Gy X-ray. Two hours after irradiation, 100 m5S cells were seeded per 100 mm dish and incubated for a week in DMEM supplemented with 10% exosome-depleted fetal bovine serum (SBI), 100 U/mL penicillin and 100 μg/mL streptomycin. For the numerical analysis of colony forming assay, 100 mm dishes were washed with PBS, fixed with 100% methanol (Wako) and stained with 5% Giemsa solution (Millipore). A colony was defined as a group of over 50 cells and colonies were counted as survivors. Colony size in 50 colonies was also measured in each condition.

To investigate MN frequency in survived cells after irradiation, 20–25 colonies of each condition were collected separately and cultured for 24 h in a 24-well plate. Trypsinized cells were then harvested and treated with 2 mL hypotonic buffer (0.1 M KCl) for 20 min at 37°C, then fixed with 2 mL ice-cold fixative (3:1 methanol:acetic acid). After centrifuging at 1200 rpm for 5 min and supernatant removal, cells were then resuspended in 2 mL fixative and incubated on ice for 5 min. After further centrifugation and supernatant removal, 1.0–1.5 mL fixative was added for cell resuspension. Cells were spread on a slide and stained with Vectashield with DAPI. MN frequency in 200 cells per colony was analyzed.

### Statistical analysis

Statistical significance of the difference between groups was assessed with tests indicated in the figure legends. StatMate III software, ver. 3.08 (ATMS) was used. If homoscedasticity was observed in the Levene’s F-test, data was analyzed by Student’s t-test. If no homoscedasticity was observed in the F-test, data was analyzed by Welch’s t-test. Statistical significance was set at *P* < 0.01 unless otherwise noted.

## RESULTS AND DISCUSSION

Firstly, ELV presence in adipose tissue culture medium was verified with immunoblot analysis of ELV markers (CD9 and CD63) (Fig. 1B). Likewise, cell uptake of PKH67-labeled ELVs was verified after 6 hours of treatment. A representative image of 4 μg/mL ELV uptake was observed in m5S cells (Fig. 1C).

**Fig. 2:**
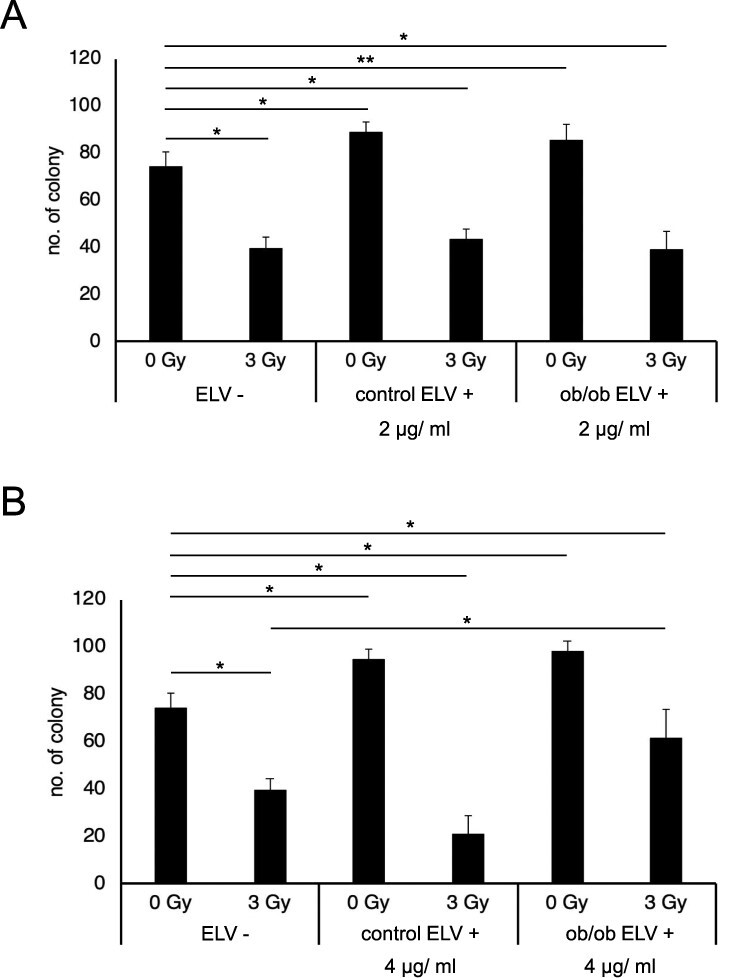
Effect of ELVs from mouse adipose on colony formation ability in m5S cells. Number of colonies in m5S cells after 0 or 3 Gy irradiation with and without 2 μg/ml ELV treatment from control and ob/ob mouse adipose tissues (ELV-, ELV+) (A), and with and without 4 μg/ml ELV treatment from control and ob/ob mouse adipose tissues (ELV-, ELV+) (B). All data are expressed as Mean ± SD (*n* = 3). *The differences were significant based on Student’s *t*-test (*P* < 0.01).

We then examined the effects of adipose ELVs on cell viability using colony formation assay. Interestingly, we observed enhanced colony formation ability in un-irradiated m5S cells after treatment of adipose ELVs (2 and 4 μg/mL) from both control mice (control ELV+) and ob/ob mice (ob/ob ELV+) (Fig. 2A and B). We have previously reported that ELVs from mouse dermal tissues (cheek and back) reduced the colony formation ability in un-irradiated m5S cells [12]. Thus, ELVs from different mouse tissues could have different effects on colony formation ability and cell viability. Compared to the untreated control (ELV-), no effect on colony forming ability was observed in 3 Gy-irradiated m5S cells after treatment of control ELV+ (2 and 4 μg/mL) or ob/ob ELV+ (2 μg/mL) (Fig. 2A and B). However, m5S cells treated with ob/ob ELV+ (4 μg/mL) showed increased survival after 3 Gy irradiation (Fig. 2B).

**Fig. 3:**
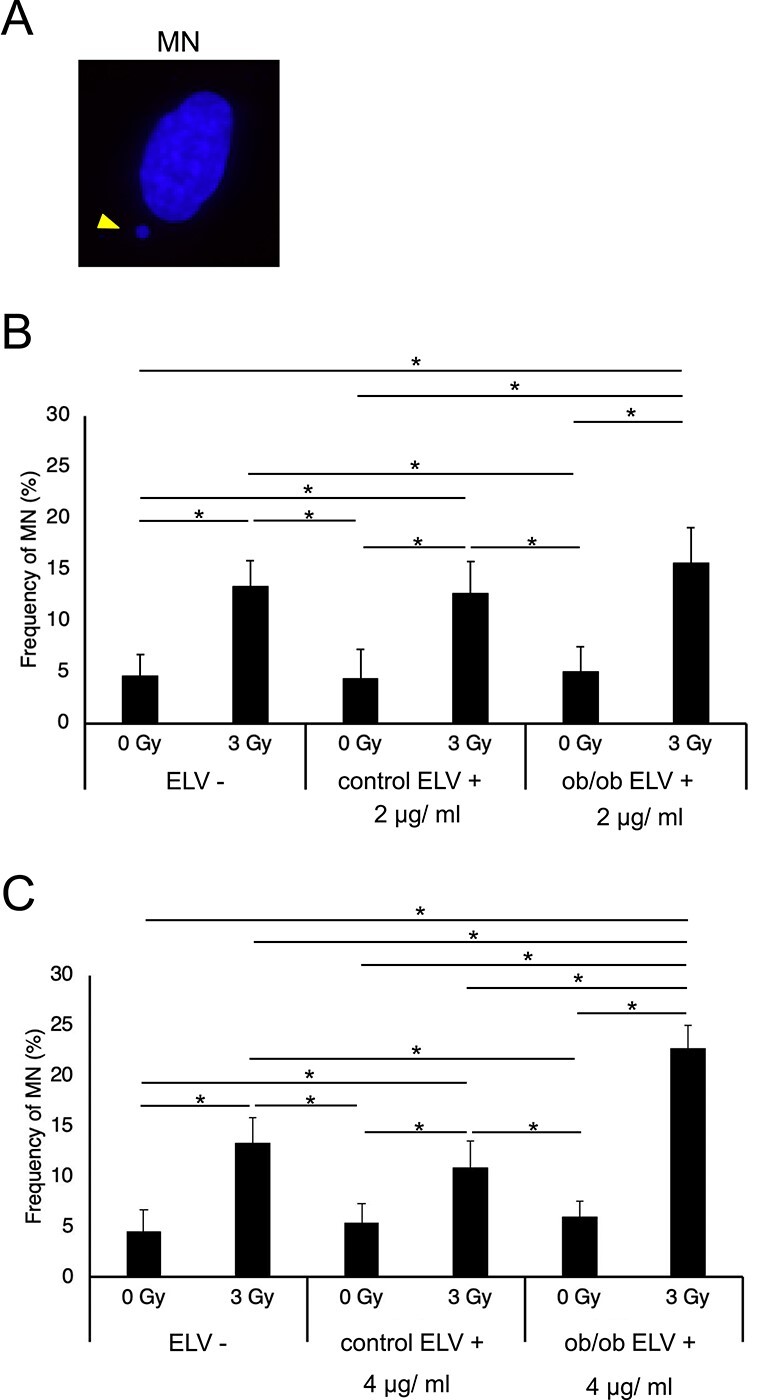
Effect of ELVs from mouse adipose on radiation-induced genomic instability measured by MN frequency in m5S cells. (A) Representative images of MN (arrowhead) in a m5S cell stained with DAPI. Scale bar: 10 μm (B) MN frequency in m5S cells after 0 or 3 Gy irradiation, with and without 2 or 4 μg/ml ELV treatment from control and ob/ob mouse adipose tissues (ELV-, ELV+). All data are expressed as Mean ± SD (n = 3). *The differences were significant based on Welch’s *t*-test (*P* < 0.01).

Next, to examine the adipose ELV effects on radiation-induced genomic instability, we analyzed MN frequency (Fig. 3A) in survived colony forming m5S cells after irradiation. While we observed increased MN frequency after 3 Gy irradiation compared to un-irradiated cells, 2 and 4 μg/mL adipose ELVs from control mice (control ELV+) and 2 μg/mL adipose ELVs from ob/ob mice (ob/ob ELV+) had no effect on MN frequency in 3 Gy-survived m5S cells (Fig. 3B, C). However, only 4 μg/mL adipose ELVs from ob/ob mice (ob/ob ELV+) increased MN in 3 Gy-survived m5S cells (Fig. 3C).

**Fig. 4:**
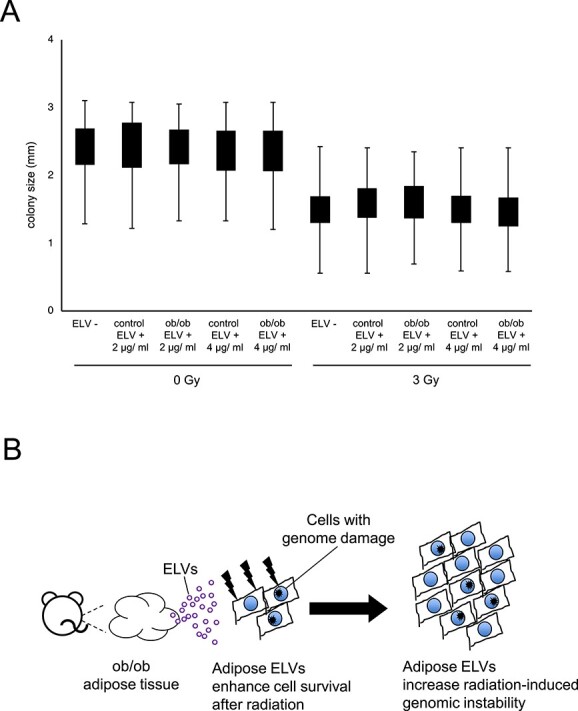
Effect of ELVs from mouse adipose on colony size and a schematic proposed model of the effect of adipose ELVs on radiation sensitivity. (A) Size of colonies after 0 or 3 Gy irradiation with and without ELV treatment (2 or 4 μg/ml) from control and ob/ob mouse adipose tissues (ELV-, ELV+). (B) Schematic proposed model of the effect of ELVs from obese mouse adipose tissues on cells with genomic instability.

Compared to the untreated control (ELV-), colony size was not affected by treatment of adipose ELV but affected by irradiation (Fig. 4A). In particular, 4 μg/mL ob/ob ELV did not affect colony size after 3 Gy irradiation, which could suggest that adipose ELV did not induce replication stress-related DNA damage in survived colonies. These results indicated that obese mouse adipose ELVs could enhance the survival of irradiated cells harboring increased radiation-induced genomic instability (Fig. 4B).

## FUNDING

This work was supported in part by JSPS KAKENHI (19K08142).

## ETHICS STATEMENT

The animal experiments were conducted according to Hirosaki University Guidelines for animal experimentation and the research protocol was approved in advance by the Ethics Committee (approval number: G17001).

## Data Availability

The datasets generated during and/or analyzed during the current study are available from the corresponding author on reasonable request.
